# Using behaviour change theory and preliminary testing to develop an implementation intervention to reduce imaging for low back pain

**DOI:** 10.1186/s12913-018-3526-7

**Published:** 2018-09-24

**Authors:** Hazel J. Jenkins, Niamh A. Moloney, Simon D. French, Chris G. Maher, Blake F. Dear, John S. Magnussen, Mark J. Hancock

**Affiliations:** 1 Department of Health Professions, Faculty of Medicine and Health Sciences, Macquarie University, Sydney, Australia; 2 Department of Chiropractic, Faculty of Science and Engineering, Macquarie University, Sydney, Australia; 3 Musculoskeletal Health Sydney, Sydney Medical School, University of Sydney, Sydney, Australia; 4 Department of Psychology, Faculty of Human Sciences, Macquarie University, Sydney, Australia; 5 Department of Clinical Medicine, Faculty of Medicine and Health Sciences, Macquarie University, Sydney, Australia

**Keywords:** Low back pain, implementation intervention, Diagnostic imaging, Intervention development, Behaviour change wheel

## Abstract

**Background:**

Imaging is overused in the management of low back pain (LBP). Interventions designed to decrease non-indicated imaging have predominantly targeted practitioner education alone; however, these are typically ineffective. Barriers to reducing imaging have been identified for both patients and practitioners. Interventions aimed at addressing barriers in both these groups concurrently may be more effective. The Behaviour Change Wheel provides a structured framework for developing implementation interventions to facilitate behavioural change. The aim of this study was to develop an implementation intervention aiming to reduce non-indicated imaging for LBP, by targeting both general medical practitioner (GP) and patient barriers concurrently.

**Methods:**

The Behaviour Change Wheel was used to identify the behaviours requiring change, and guide initial development of an implementation intervention. Preliminary testing of the intervention was performed with: 1) content review by experts in the field; and 2) qualitative analysis of semi-structured interviews with 10 GPs and 10 healthcare consumers, to determine barriers and facilitators to successful implementation of the intervention in clinical practice. Results informed further development of the implementation intervention.

**Results:**

Patient pressure on the GP to order imaging, and the inability of the GP to manage a clinical consult for LBP without imaging, were determined to be the primary behaviours leading to referral for non-indicated imaging. The developed implementation intervention consisted of a purpose-developed clinical resource for GPs to use with patients during a LBP consult, and a GP training session. The implementation intervention was designed to provide GP and patient education, remind GPs of preferred behaviour, provide clinical decision support, and facilitate GP-patient communication. Preliminary testing found experts, GPs, and healthcare consumers were supportive of most aspects of the developed resource, and thought use would likely decrease non-indicated imaging for LBP. Suggestions for improvement of the implementation intervention were incorporated into a final version.

**Conclusions:**

The developed implementation intervention, aiming to reduce non-indicated imaging for LBP, was informed by behaviour change theory and preliminary testing. Further testing is required to assess feasibility of use in clinical practice, and the effectiveness of the implementation intervention in reducing imaging for LBP, before large-scale implementation can be considered.

**Electronic supplementary material:**

The online version of this article (10.1186/s12913-018-3526-7) contains supplementary material, which is available to authorized users.

## Background

Low back pain (LBP) is a common problem, with a mean one-year prevalence of 38.1% [1]. It is one of the leading causes of global disability [2] and care seeking [3], and is associated with high direct (medical) and indirect (non-medical) costs [4], resulting in large economic and social burden.

Diagnostic imaging, such as x-ray, CT, or MRI, is commonly used to investigate LBP but has limited utility. Imaging is only indicated in cases of suspected serious pathology (e.g. cancer or infection), or cases of specific pathology (e.g. spinal stenosis) where surgery is being considered [4]. These are estimated to account for less than 10% of all LBP presentations [3, 4]. For other LBP presentations, imaging has not been shown to improve clinical outcomes and is associated with unnecessary radiation exposure, increased costs to the patient and healthcare system, and potentially inappropriate treatment [5]. Although clinical practice guidelines recommend imaging only in certain cases of LBP, poor adherence to these guidelines is seen in clinical practice [4, 6, 7].

Overuse of imaging for LBP has been identified as a problem in general medical practice [5, 8, 9], with between one-third to one-half of requested imaging considered inappropriate [10–14]. Many potential barriers to reducing imaging for LBP have been reported, including both practitioner and patient-related factors [15]. Interventions aiming to address practitioner-related barriers have been assessed, including guideline dissemination, practitioner education, audit and feedback of imaging practices, and clinical decision support [16]. Only clinical decision support demonstrated evidence of effectiveness [16], however, this can be difficult to implement in general medical practice.

A large proportion of patients believe that imaging is important for the correct diagnosis and management of LBP [17–19]. This belief has been associated with increased imaging referrals [20, 21], and therefore may be an important barrier to address. Several studies have investigated population-based education interventions aiming to change beliefs about back pain, with varying results on the use of imaging [22–25]. Individualised patient education has been shown to improve general back pain beliefs [26, 27], however, whether individualised patient education is an effective intervention to reduce imaging for LBP has not been studied [16]. The development of an effective intervention, addressing both practitioner and patient related barriers to reducing non-indicated imaging, which can be successfully implemented in clinical practice, would be of great public health value.

The process of designing effective interventions to change behaviour in clinical practice is challenging. Process models, including the Behaviour Change Wheel [28, 29] and the Theoretical Domains Framework [30], have been developed to guide the development of implementation interventions to facilitate behaviour change [31]. These typically incorporate elements of: 1) analysis of the underlying behaviour; 2) selection of appropriate intervention techniques; 3) design of an implementation strategy; and 4) evaluation of the developed intervention [32–34]. Previously developed interventions to improve LBP care and reduce inappropriate imaging have generally not used an underlying theoretical framework [31, 35].

Preliminary testing of developed interventions is important to improve implementation of the intervention within clinical practice [33]. Identification of barriers and facilitators to the implementation of developed interventions is rarely conducted [31], potentially reducing the effectiveness and impact of the intervention in a clinical setting.

The aim of this study was to develop an implementation intervention aiming to reduce non-indicated imaging for LBP, by targeting both general medical practitioner (GP) and patient barriers concurrently.

## Methods

### Overview of development and preliminary testing of the implementation intervention

**Fig. 1:**
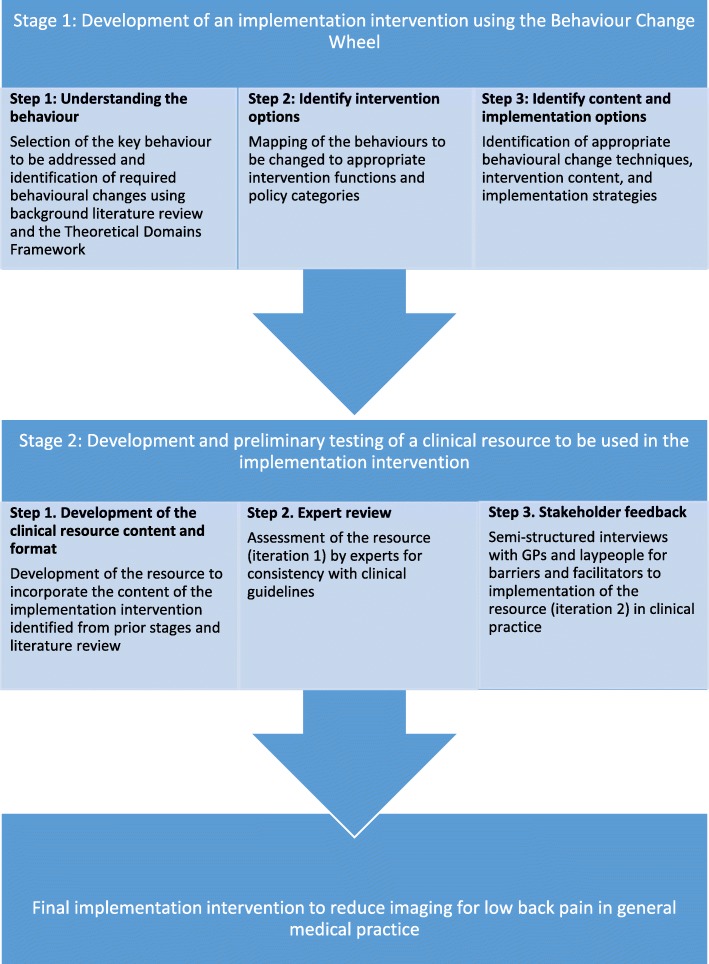
Process of developing an implementation intervention to reduce imaging for low back pain

Figure 1 outlines the process used to develop, and perform preliminary testing of, an implementation intervention to reduce GP referral for non-indicated imaging in the management of LBP using the Behaviour Change Wheel [28, 29], with integration of the Theoretical Domains Framework [28, 30]. Ethics approval was granted by Macquarie University Human Research Ethics Committee (MUHREC), reference number: 5201600298.

#### Stage 1: Development of an implementation intervention using the behaviour change wheel

The three steps of the Behaviour Change Wheel [28], as depicted in Fig. 1, were initially completed by one author. To perform an in-depth analysis of the behaviours to be changed, barriers and facilitators to reducing imaging for LBP were identified through literature review. The APEASE criteria as defined in the Behaviour Change Wheel (Affordability, Practicability, Effectiveness and cost-effectiveness, Acceptability, Side-effects and safety, Equity) were considered to direct the selection of appropriate intervention options, content, and implementation options [28]. Discussion among all authors was used to arrive at a consensus of a draft implementation intervention that was considered to be appropriate, practical and economical within a primary care setting.

#### Stage 2: Development and preliminary testing of a clinical resource to be used in the implementation intervention

In stage 1, it was determined that development of a purpose-designed clinical resource would be required to incorporate identified intervention content and implementation strategies. This clinical resource would be a LBP management and education booklet, to be used by GPs during a clinical consult, to improve the GPs ability to manage LBP without referring for non-indicated imaging, while simultaneously reducing pressure from the patient to refer for imaging. Development and preliminary testing of the resource was performed as described in Fig. 1. A review of the literature was used to identify: 1) key educational messages to be incorporated into the resource; 2) patient perspectives on the management of LBP and what information they wish to receive; and 3) evidenced-based management strategies for LBP. The draft resource was sent for design and marketing feedback to optimise visual impact and readability.

##### Expert review of the clinical resource (iteration 1)

The first iteration of the developed clinical resource was sent for assessment to five international LBP experts, including radiologists, rheumatologists, and general medical practitioners. They were asked to complete a written questionnaire asking: 1) if the information in the resource was consistent with current guidelines; 2) if they thought use of the resource would be likely to change behaviour; and 3) if the information was provided in a suitable format. Questionnaire responses were summarised and the resource was modified based on these responses, after discussion and consensus from all authors, to develop a second iteration of the clinical resource.

##### Stakeholder feedback on the clinical resource (iteration 2) and its proposed implementation into clinical practice

Stakeholder feedback was sought through semi-structured interviews from GPs and health consumers (laypeople with a history of LBP) to identify barriers and facilitators to implementation of the clinical resource in clinical practice. GPs and health consumers were recruited from Sydney (and surrounding areas), New South Wales, Australia.

Convenience sampling of GPs was performed until thematic saturation was reached. To be included, GPs needed to be in current practice and seeing patients with LBP. GPs were sampled to include a range of gender, years of experience, and practice location in different socioeconomic areas.

Health consumers were recruited through advertisements in print format and on social media until thematic saturation was achieved. To be included, laypeople needed to be over the age of 18, have a history of LBP, and be able to read and understand English. Sampling was conducted to ensure a range of gender, ages, and cultural and educational backgrounds.

All participants were provided with a copy of the second iteration of the clinical resource and asked to read it before participating in an audio-recorded interview with one of the authors. Participants received an AUD$30 gift voucher for their time. Interview questions included: background demographic questions; current beliefs about imaging for LBP; barriers and facilitators to implementation of the resource in clinical practice; appropriateness of the included information; and whether use of the resource would be likely to change behaviour.

Interviews were transcribed and coded by one author. Thematic analysis using the Theoretical Domains Framework [30] was initially performed by one author, with iterative review and discussion from other authors, until final themes and potential changes to the resource and its implementation were determined.

### Final implementation intervention to reduce imaging for LBP in general medical practice

The draft implementation intervention was revised based on results from preliminary testing. Potential changes to the implementation intervention were discussed with all authors before final changes to the implementation intervention components (including the clinical resource) were made.

## Results

### Stage 1: Development of a draft implementation intervention using the behaviour change wheel

#### Step 1: Understanding the behaviour

**Fig. 2:**
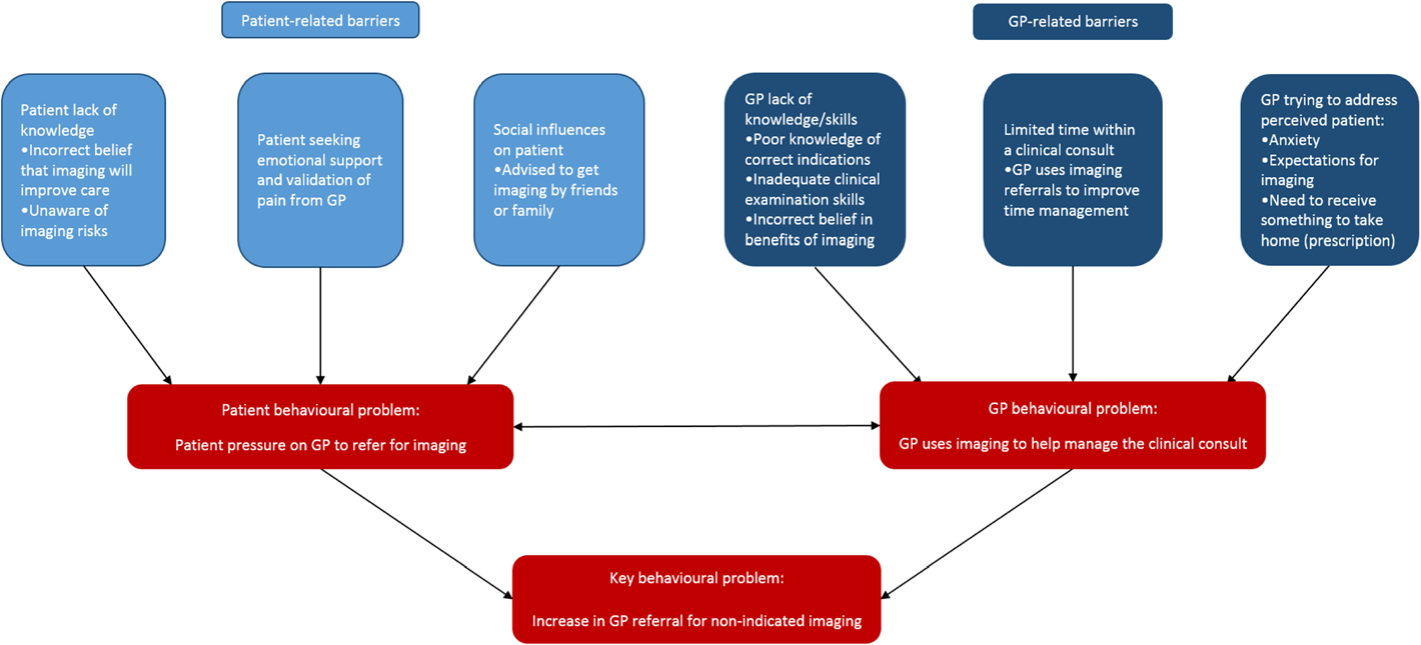
Concept map of the identified barriers to reducing imaging for low back pain

The behavioural problem to be addressed was defined by the authors as: GPs referring for non-indicated imaging in patients presenting with LBP. LBP was not restricted to type (i.e. acute or chronic) or whether the patient had received prior management. Instead the focus was on any presentation of non-specific LBP where imaging was not indicated. Barriers and facilitators to reducing GP referral for non-indicated imaging were identified through literature review, and are outlined in Table 1. Figure 2 depicts a concept map of how the identified barriers are likely to drive an increase in GP referral for non-indicated imaging. Patient-related barriers are likely to increase the likelihood of a patient requesting imaging from the GP. GP-related barriers are likely to increase the likelihood of the GP using imaging to help manage the LBP consult. The interaction between the patient and GP behaviours during a clinical consult is likely to increase GP referral for non-indicated imaging. Therefore, both patient and GP behaviours during a clinical consult need to be addressed concurrently in the implementation intervention.

**Table 1:** Changes required at the general practitioner (GP) and patient level to reduce GP use of non-indicated imaging for low back pain, mapped to the associated barriers and facilitators, the domains of the Theoretical Domains Framework, and the Behaviour Change Wheel

Changes required to reduce referral for non-indicated imaging for low back pain	Barriers and facilitators (identified through literature review) that will be influenced by the identified change	Theoretical Domains Framework component	COM-B component (Behaviour Change Wheel)
General practitioner (GP) changes required:			
- GPs need to have the skills to: 1. Screen for clinical suspicion of underlying pathology to determine if imaging is necessary 2. Communicate with patients to explain their diagnosis and advise them that they don’t need imaging	Barriers: - Diagnostic uncertainty [30, 46, 54, 55] GPs uncertain in their skills in adequately diagnosing low back pain without imaging; Fear of missing a diagnosis of underlying pathology - Unsure how to advise patients that imaging is not needed [52] GPs uncertain how to convincingly explain to patients that imaging is not needed Facilitators: - Communication with patients [46] GPs confident in communicating with patients, to educate and reassure them	Skills	Physical capability
- GPs need to have knowledge of: 1. Guidelines and appropriate indications for imaging 2. Limitations of imaging in the diagnosis and management of low back pain 3. Risks of imaging 4. Key concepts required in patient explanations explain why imaging isn’t necessary	Barriers: - Lack of guideline awareness [30, 46, 52, 55] GPs lack knowledge and awareness of current guidelines recommending appropriate use of imaging for low back pain - Unsure how to advise patients that imaging is not needed [52] GPs uncertain how to convincingly explain to patients that imaging is not needed Facilitators: - Guideline awareness [51, 52] GPs display knowledge of current guidelines recommending appropriate use of imaging for low back pain - Awareness of limitations of imaging [51] GPs aware of limitations of imaging in providing diagnoses, directing management, or reassuring patients. - Awareness of danger of radiation exposure [51] GP aware that x-rays and CT scans add to radiation exposure and may be harmful	Knowledge	Psychological capability
- GPs need to use a decision-making process which incorporates the appropriate use of imaging	Barriers: - Diagnostic uncertainty [30, 46, 54, 55] GPs uncertain in their skills in adequately diagnosing low back pain without imaging; Fear of missing a diagnosis of underlying pathology Facilitators: - Availability of guidelines [51] Guidelines act as a memory-aid and are more likely to be followed if they are accessible, concise and user-friendly.	Memory, attention, and decision process	Psychological capability
- GPs need to have: 1. Increased time for patient education 2. Cues to remind them of imaging appropriateness 3. Resources to give to patient to improve ability to educate and reassure the patient in a limited time	Barriers: - Time constraints [30, 48–50, 54, 55] GPs don’t have enough time with patients to provide explanations and reassurance; Imaging seen as a quick way to reassure the patient and increase patient compliance - Diagnostic uncertainty [30, 46, 54, 55] GPs uncertain in their skills in adequately diagnosing low back pain without imaging; Fear of missing a diagnosis of underlying pathology - Perceived need to give the patient something to take home [30] GPs feel that patients expect to receive something from the consult and an imaging referral is often used to achieve this	Environmental context and resources	Physical opportunity
- GPs need to use their role as a trusted source of information provision to educate patients	Facilitators: - Communication with patients [46] GPs confident in communicating with patients, to educate and reassure them - Senior GP who adheres to guidelines [52] Having a senior GP to model correct behaviour and act as a potential opinion leader to the other GPs	Social influences	Social opportunity
- GPs need to be confident in their ability to: 1. Screen for clinical suspicion of underlying pathology to determine feel that imaging Ps feel that imaging if imaging is necessary 2. Reassure patients without imaging	Barriers: - Perceived patient expectations [30, 46, 48, 50–52, 54, 55] GPs feel that patients often want or expect imaging, and that they don’t understand the limited usefulness of imaging to manage low back pain; Fear that patients will be upset if they don’t receive imaging or may devalue the GP	Beliefs about capabilities	Reflective motivation
- GPs need to be aware of the risks and benefits of referring for imaging, and the likely consequences of referring for imaging when it isn’t indicated	Barriers: - Perceived usefulness of imaging and negative consequences to following guidelines [30, 46, 47, 49–52, 55] GPs feel that imaging will be useful – provide diagnosis, help to reassure the patient, help to facilitate patient management, build patient relationships; They feel there are more negative consequences associated with following guideline advice not to refer for imaging - Pressure from patients [20, 49–51, 54, 55] GPs report that they receive direct pressure from patients to refer for imaging; They feel that if they don’t comply with the request patients will devalue them and go elsewhere to obtain imaging - Perceived patient anxiety [30, 46, 47, 49, 51, 55] GPs perceive that imaging will help to reassure anxious patients that their condition is not serious and will increase compliance with advice - Possible litigation [48, 51, 55] GPs feel that they may open themselves to possible litigation if they don’t refer for imaging - Specific patient characteristics [46, 50] Specific patient characteristics more likely to lead to increased imaging (i.e. elderly, workers compensation claims, etc.) Facilitators: - Perceived positive consequences to following guidelines [30, 55] GPs are in agreement with the guidelines and feel that more positive consequences are associated with following guideline advice not to refer for imaging	Beliefs about consequences	Reflective motivation
Patient changes required:			
- Patients need to have knowledge of: 1. Limitations of imaging in the management of low back pain 2. Risks of imaging 3. Signs to be aware of that may indicate the need for imaging	Barriers: - Perceived reassurance and explanation of symptoms from imaging [19, 21, 45, 56] Patients feel that imaging will provide reassurance to them by excluding pathological causes of low back pain and providing a diagnosis, particularly when pain levels are high or not resolving - Lack of awareness of risks of imaging [19] Patients report being unaware of potential risks of imaging, and even where some risks are recognised report that potential benefits outweigh these risks.	Knowledge	Psychological capability
- Patients need to be aware of the decision process that was used to determine that they don’t need imaging	Barriers: - Perceived reassurance and explanation of symptoms from imaging [19, 21, 45, 56] Patients feel that imaging will provide reassurance to them by excluding pathological causes of low back pain and providing a diagnosis, particularly when pain levels are high or not resolving Facilitators - Communication with patients [46] Patients whose GPs communicate with them adequately are more likely to be reassured without the use of imaging	Memory, attention, and decision process	Psychological capability
- Patients need to receive educational resources focusing on patient reassurance, appropriate management and why imaging isn’t required	Barriers: - Perceived reassurance and explanation of symptoms from imaging [19, 21, 45, 56] Patients feel that imaging will provide reassurance to them by excluding pathological causes of low back pain and providing a diagnosis, particularly when pain levels are high or not resolving - Lack of awareness of risks of imaging [19] Patients report being unaware of potential risks of imaging, and even where some risks are recognised report that potential benefits outweigh these risks	Environmental context and resources	Physical opportunity
- Patients need to have less access to contradictory information sources, or more access to evidence-based information sources	Barriers: - Influences from friends, family, or other healthcare practitioners, and previous experience that imaging is important [19] Advice from friends, family, or other healthcare practitioners, and previous experience of referral for imaging for low back pain likely to increase perceived need for imaging	Social influences	Social opportunity
- Patients need to be aware of possible outcomes of the suggested management plan, and possible consequences of being referred for imaging when not indicated	Barriers: - Perceived reassurance and explanation of symptoms from imaging [19, 21, 45, 56] Patients feel that imaging will provide reassurance to them by excluding pathological causes of low back pain and providing a diagnosis, particularly when pain levels are high or not resolving	Beliefs about consequences	Reflective motivation
- Patients need to feel that they are receiving emotional support from the GP without imaging	Barriers: - Emotional support and validation of pain from GP referring for imaging [21] Patients feel that GPs who comply with their wishes to refer for imaging are providing necessary emotional support and validating their pain	Emotion	Automatic motivation

Table 1 lists the specific changes required in GPs and patients to decrease GP referral for non-indicated imaging based on the identified barriers and facilitators. These changes were mapped to capability, opportunity, and motivation components (COM-B model) from the Behaviour Change Wheel and to domains from the Theoretical Domains Framework that need to be considered to bring about a change in behaviour (Table 1).

#### Step 2: Identify intervention options

Using the Behaviour Change Wheel [28], suitable intervention options were identified from nine intervention functions (means by which an intervention will change behaviour) and seven policy categories (means by which an intervention will be delivered) as presented in Additional file 1.

The intervention functions that met the APEASE criteria were: Training; Education; Environmental restructuring; Enablement; Modelling; and Persuasion. Although education alone (such as guideline dissemination, or provision of information) has not shown evidence of effectiveness [16], it was decided that it was important to include this intervention function to address the domain of ‘Knowledge’ from the Theoretical Domains Framework. The combination of education with the other identified intervention functions was hypothesised to be more effective than education alone. Clinical decision support and regular reminders of correct indications for imaging have shown evidence of effectiveness at reducing imaging for LBP [16] and are, therefore, important to include in the implementation intervention through environmental restructuring.

The policy categories that met the APEASE criteria were: Service provision; Communication/marketing; and Environmental/social planning. Regulations and guidelines around the appropriate use of imaging currently exist, and rather than develop new guidelines, the aim of the developed implementation intervention is to increase current guideline adherence. Changes to fiscal measures and legislation are outside the ability of the research team, and may lead to issues with acceptability and safety if clinical decision-making is made too restrictive.

#### Step 3: Identify content and implementation options

Results from the prior stages of the Behaviour Change Wheel were used to guide selection of appropriate behavioural change techniques, and the resultant content and mode of delivery of the implementation intervention, as presented in Table 2.

**Table 2:** Mapping of the intervention function (means by which an intervention will change behaviour), to behaviour change technique, and to content and mode of delivery of the draft implementation intervention

Intervention function (targeted to GP/Patient)	Behavioural change technique	Implementation intervention: content	Policy category	Implementation intervention: mode of delivery
Education (GP)	Information about health consequences	Guidelines for appropriate diagnosis and management of low back pain	1. Communication/marketing 2. Service provision	1. Providing GP with educational material - Copies of current guidelines provided to GP [4, 38] 2. Training session with GP - Verbal discussion of guidelines
		Information regarding the appropriate diagnosis and management of low back pain	1. Communication/marketing	1. Providing GP with educational material - Copies of developed clinical resource provided to GPs to read
	Prompts/cues	Decision tree for appropriate imaging for low back pain (clinical decision support)	1. Environmental/social planning	1. Providing GP with clinical resources - Copies of developed clinical resource provided to GPs to use during a consult, includes decision tree for clinical decision support
		Management plan	1. Environmental/social planning	1. Providing GP with clinical resources - Copies of developed clinical resource provided to GPs to use during a consult, includes customisable management plan
Training (GP)	Feedback on the behaviour	Explanation of the goals of using the clinical resource to reduce imaging for low back pain	1. Communication/marketing 2. Service provision	1. Providing GP with training material - Information sheet about the developed clinical resource provided to GPs to read 2. Training session with GP - Verbal discussion of goals
	Instruction on how to perform a behaviour	Instruction on how the developed clinical resource can be used: - as clinical decision support - as a checklist or reminder of correct management - to provide key educational messages to patients - to provide individualised management advice - in a time-efficient manner	1. Communication/marketing 2. Service provision	1. Providing GP with training material - Information sheet about the developed patient education booklet provided to GPs to read 2. Training session with GP - Verbal discussion of how to use the developed clinical resource
Modelling (GP)	Demonstration of a behaviour	Modelling of appropriate information to be given to the patient during a consult	1. Environmental/social planning 2. Service provision	1. Providing GP with clinical resources - Copies of developed clinical resource provided to GPs to use during a consult, includes key messages to be delivered to patient 2. Training session with GP - Demonstration by training facilitator of how to use the developed clinical resource
Environmental restructuring and Enablement (GP)	Adding objects to the environment	Developed clinical resource for use during a consult - Facilitate GP-patient communication - Provide a tool to help educate and reassure patients during a consult, in a time-efficient manner - Provide clinical decision support, and a reminder of appropriate imaging use and management advice to give to patient	1. Environmental/social planning	1. Providing GP with clinical resources - Copies of developed clinical resource provided to GPs to use during a consult
Education (Patient)	Information about health outcomes	Information to: - Address common misconceptions around low back pain, with a particular focus on imaging - Reassure the patient that their low back pain is not serious - Explain why imaging is not necessary - Provide suitable management advice - Provide information regarding symptoms associated with more serious pathology	1. Communication/marketing	1. Providing patient with educational material - GP delivers the developed clinical resource to the patient during a consult, providing key messages and individualising the management plan - Patient can use the resource as an ongoing resource of information and individualised management advice
Persuasion (Patient)	Credible source	Clinical resource delivered by GP and developed by a reputable university research team	1. Environmental/social planning	1. Providing patient with clinical resources - GP delivers the developed clinical resource to the patient during a consult, providing key messages and personalising the management plan
	Information about health consequences	Decision tree for appropriate imaging for low back pain (clinical decision support)	1. Service provision	1. GP-Patient consult - GP uses the decision tree in the clinical resource during the consult to explain to the patient why they don’t need imaging, facilitates shared decision making
Environmental restructuring and Enablement (Patient)	Adding objects to the environment	Customisable clinical resource given to patient in consult - Facilitate GP-patient communication - Short, appealing and easy to read with limited text and clear information - Reinforce or remind of information provided within the consult - Provide appropriate, individualised management advice - Provide links to other resources with guideline consistent messages	1. Environmental/social planning	1. Providing patient with clinical resources - GP delivers the developed clinical resource to the patient during a consult, providing key messages and personalising the management plan - Patient can use the booklet as an ongoing resource of information and individualised management advice

It was determined that a clinical resource for GPs to use within the clinical consult would be required to facilitate delivery of the content of the implementation intervention to both GPs and patients. The clinical resource would be designed to facilitate GP-patient communication. The resource would: 1) provide clinical decision support; 2) act as a reminder to the GP of correct indicators for imaging; 3) facilitate GP communication with the patient by providing key messages to be delivered to the patient during a consult (e.g. explaining clinical reasoning for not using imaging); 4) provide customisable management strategies to be delivered to the patient; and 5) be sent home with the patient to act as a management ‘prescription’ and an ongoing educational resource for LBP.

**Fig. 3:**
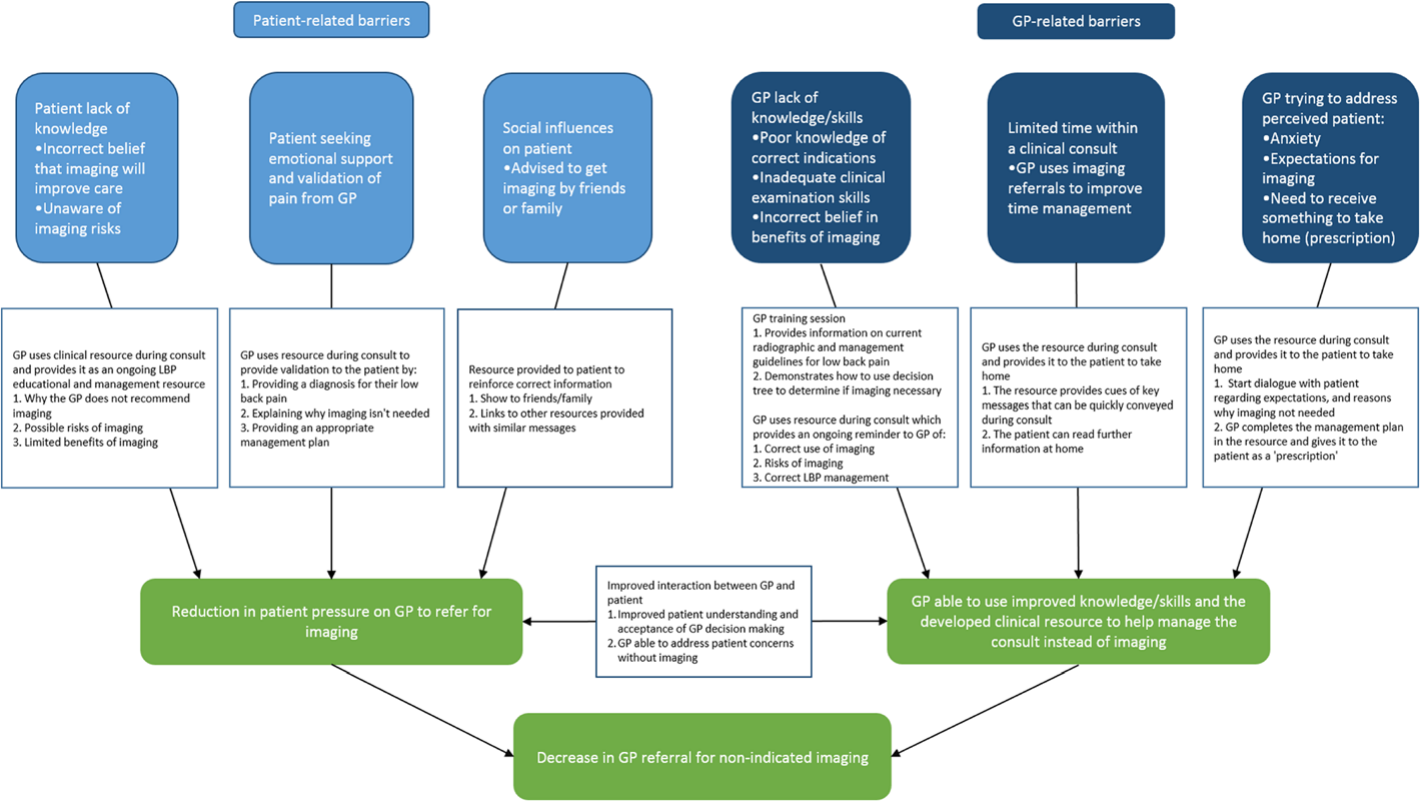
Concept map of how the implementation intervention will target identified barriers

A GP training session was included in the implementation intervention to: 1) provide GP education on indicators for imaging for LBP; and 2) explain and demonstrate the integration of the clinical resource into a LBP consult. Figure 3 depicts how the intervention components have been designed to address the identified barriers, and improve GP and patient behaviours.

### Stage 2: Development and testing of the clinical resource

#### Development of the clinical resource content and format

Currently available LBP clinical resources were assessed for inclusion in the draft implementation intervention. It was determined that a purpose-designed resource would be necessary to incorporate the content of the implementation intervention, and facilitate mode of delivery (Table 2). In particular, the resource needed to: 1) be a tool that the GP could work though with the patient in a time-efficient manner; 2) include clinical decision support and key educational messages; 3) include customisable management advice; and 4) be appealing, quick and easy for the patient to read after the consult. The developed clinical resource was a LBP management and education booklet that could be individualised to the patient.

The clinical resource content was developed using LBP guidelines [36–38], review articles [4, 39], and other educational resources [40–43]. Key messages to deliver to patients were identified through literature review of qualitative studies providing patient feedback on LBP management [44, 45], while always considering if these were likely to help reduce patients’ desire to receive imaging. The first iteration of the clinical resource included:

1. A decision tree, based on diagnostic triage, for the GP to complete to provide clinical decision support for the GP, and facilitate GP-patient communication to demonstrate why imaging is not required
2. Information on: LBP and common causes; why imaging usually isn’t necessary; and what the patient can do to help their LBP
3. A customisable LBP management plan for the GP to complete, including advice to stay active, simple pain management strategies, information on what to do if the pain does not resolve, and symptoms that may indicate need to return to the GP
4. Links to further evidence-based resources about LBP

#### Expert review of the first iteration of the clinical resource

All five experts initially approached consented to be involved in the study. All experts reported that the key messages and specific content within the clinical resource were consistent with current knowledge and published guidelines. Potential barriers to the use of the resource, or its ability to change behaviour were suggested, including: confusion regarding the intended audience: whether it was designed to educate GPs or patients; complexity of some of the language used, potentially limiting patient understanding; and the time the GP would need to explain the resource to the patient.

#### Resultant changes to the first iteration of the clinical resource

Changes to specific wording were adopted to: 1) increase clarity to show that the intended audience was the patient; 2) simplify the language; and 3) highlight messages of patient reassurance and the limitations of imaging.

#### Stakeholder feedback on the second iteration of the clinical resource

Thematic saturation was reached with the recruitment of ten GPs and ten health consumers. Of the GPs, six were female, two had a special interest in LBP, and they had a mean of 12.4 years in clinical practice (range: 1–30 years). Of the health consumers, five were female, seven had a university level education, seven came from a Caucasian cultural background, and the average age was 41.4 years (range: 30–65 years). Nine of the health consumers had previously received imaging for LBP.

##### Content and format of the clinical resource

Both GPs and health consumers agreed that the general content and layout of the developed resource were appropriate, that it included important and useful information, and was appealing to read. Some wording was identified as potentially confusing. For example, ‘specific cause of low back pain’ was interpreted by some to refer to the mechanism of action causing the LBP (e.g. lifting), rather than as an underlying pathology (e.g. infection) as intended. It was identified that the management plan in its current format would only be useful to the patient if completed by the GP, which may not always be possible. Some GPs raised concerns that the use of ice as a treatment strategy was not in line with their clinical practice. Finally, the links to additional resources were reported to be too small to read, and the website addresses were too long to easily use.

##### Barriers and facilitators to implementation of the developed clinical resource in clinical practice

Specific barriers and facilitators to implementation of the clinical resource by GPs and health consumers were identified and are presented in Additional file 2.

Hardcopy format of the clinical resource:

Barriers to the use of the clinical resource as a hardcopy booklet were identified by GPs, including: potential purchasing costs; recency of included information; and difficulty following electronic links. Electronic formats, in particular A4 formatted handouts, which could be printed out for the patient, were preferred by most GPs. Some GPs were also happy to use email or website options, however, others reported that they would be unlikely to use these.

Conversely, most health consumers found the clinical resource as a hardcopy booklet a facilitator of use, reporting that they would be more likely to keep and continue referring to a booklet whereas printed handouts were often thrown away. Email or website options were not preferred as they would forget to look at them, although it was recognised that the links to other online resources would be easier to follow from these.

Usefulness of the clinical resource:

Most GPs and health consumers reported that they would find the resource useful to either use in clinical practice or to receive. GPs felt that LBP can be difficult to manage and resources are needed. They also reported that patients seem more satisfied if they receive something to take home with them. The few GPs who said they were unlikely to find the resource useful reported that they didn’t feel much pressure to refer for imaging and didn’t require added resources.

Health consumers thought that the information in the resource was relevant and important to them, and that it would help to reinforce the GPs opinion and advice. Some GPs felt that the resource wouldn’t be useful with all patients, and that they would be more likely to use the resource with patients needing further reassurance or explanations. Health consumers also felt that receiving the resource wouldn’t always be appropriate, such as when imaging was indicated, or if they were experiencing high pain levels.

Use of the clinical resource in a LBP consult:

A commonly reported barrier to the use of the resource from GPs was the ability to conveniently store a hardcopy booklet and remember to use it. Most GPs preferred an electronic option that could be stored on the computer. There was some concern that using the resource might be time-consuming for a standard consult, but others felt that it would aid time management. Health consumers felt that the resource was time-efficient to read and easy to refer to.

GPs provided different suggestions on how the resource could be used in clinical practice. These included: 1) as a reminder for themselves of correct management; 2) as an explanatory aid during the consult to explain the LBP diagnosis and management to the patient, and explain why imaging is not necessary; 3) filling in the resource to provide the patient with an individualised management plan; and 4) as an educational resource for the patient to take home.

Health consumers said they were more likely to use the resource if it had been individualised to them, and that it would be most useful if the information was reinforced by the GP taking them through it. Health consumers reported they would be likely to continue using the resource to remind them of appropriate LBP management.

##### Perceived likelihood of the clinical resource to decrease imaging for LBP

Themes related to whether the clinical resource (iteration 2) would be likely to help reduce imaging for LBP are presented in Table 3.

**Table 3:** Themes from qualitative interviews on possible change in behaviour with use of the clinical resource (iteration 2)

General practitioner	
Booklet would help to decrease imaging pressure from patients “Yeah [help decrease pressure felt to refer patients for imaging] because I mean it’s got the resources, the references at the back and the websites that they can look up for more information to see why it’s not necessary, rather than just coming from my word of mouth” (GP10) Booklet provides a reminder of correct imaging decisions for the GP “…[algorithm] also helpful for the doctor as a reminder maybe for someone who doesn’t, just as a reminder you know, think of those sort of red flags that need to be screened for” (GP8) Potential negative consequences of not referring for imaging when a patient really wants it “if people are hell-bent on getting imaging you’re pretty dumb not to give it to them, because it’ll be the one that you don’t that’ll be the one that has some bizarre weird tumour or something” (GP5) “I think if someone was adamant that they wanted an x-ray I think that they would be unhappy leaving the room without an x-ray request form, whether you give them this paperwork or not” (GP10)	
Health consumers	
Information in the booklet is reassuring “I found it quite reassuring you know that quite a lot of people who have imaging might show up you know some kind of change which isn’t actually going to be problematic in terms of effect to their life” (MoP2) “I guess it’s reassuring to know that everyone will get back pain, or most people will get back pain, but the what you can do to decrease it is super helpful” (MoP6) Useful to receive the booklet from the GP to give appropriate information and management “I think it would be helpful [to receive the booklet from the GP] because I think different people approach GPs with a different pace of knowledge and different set of expectations” (MoP1) “it [having the GP go through the booklet] highlights that you are going through and thinking about it and that you’ve got a booklet telling you the same thing and a GP telling you the same thing which sort of reinforces the message” (MoP1) “I should think so [feel reassured]. I know I mean again a lot of people are different but I think the fact that you’re getting it through the GP I think for a lot of people that gives it extra credibility” (MoP3) Booklet demonstrates why the GP made their decision not to refer for imaging “that little the thing on page 2 [flowchart] makes it very clear on which way, which pathway you need to go basically” (MoP8) Booklet provides a reminder of management advice “I think it’s good that GPs told me things but I might get distracted by other things happening in life as well, so if I had a booklet I could always refer back and so it’s like a dictionary – if I need to look up something I can always refer to this booklet” (MoP5) Booklet can be used to by patients to monitor their progress and when they need to go back to the GP “If you haven’t been to the doctor for a while and you think hang on what should I do again, like what should I do, should I go back - that whole when should I return for further medical advice [in the booklet] that I think that’s really good” (MoP4) “Yes [would feel reassured back pain being managed correctly]; that sort of makes you feel that you know what to do if it gets worse. So you know it’s been managed at the level it’s at and then if it gets any worse you can look here and go, oh yeah, that happens, probably should go get that checked” (MoP7) Reading booklet changed beliefs on the importance of imaging “I think a lot of people believe, and I certainly believed, that this [imaging] would give you that answer” (MoP1) “[the booklet states] that you should always look to solve pain with the least amount of surgery, doctors, x-rays, things as possible first” (MoP4) “I do think it [the booklet] would have changed the way I thought about imaging at first” (MoP2) “Yeah, yeah for sure [booklet change beliefs]. Now I know that imaging won’t necessarily show anything or it will only show something that most people will also have but not necessarily have pain for. I didn’t know that at all” (MoP7) Booklet unlikely to change beliefs on the importance of imaging “Not to me [booklet help change beliefs], I think, I would still, I would still get an x-ray or something at the start just to make sure” (MoP9) “I believe in a pain threshold if it’s really painful then generally it’s a sign something serious is wrong so that then you should probably consider getting imaging more strongly” (MoP1)	

GPs reported that they thought that using the resource would be likely to facilitate appropriate imaging decisions by decreasing pressure from patients to refer for imaging. They also felt it would provide a useful reminder of correct imaging decisions for themselves. Some GPs did feel that there may be negative consequences of not imaging if a patient really wanted it.

Health consumers reported that the information in the resource was likely to make them more accepting of the GP decision not to image by: 1) reassuring them about the generally benign nature of LBP and why imaging is unnecessary; 2) being able to see why the GP made their decision; and 3) providing management and follow-up advice they could keep referring to. Some health consumers reported that reading the resource alone had provided an adequate explanation of why imaging wasn’t always necessary, and they would be less likely to think imaging was necessary in the future. Conversely, some health consumers reported that they still believed imaging to be necessary to ensure no serious pathology was present, or in situations where they were experiencing high pain levels, despite reading the resource.

#### Resultant changes to the second iteration of the clinical resource

Changes to the clinical resource from the aforementioned stages included: 1) changes to wording; 2) modification of the management plan; and 3) changes to the presentation of website links. A PDF copy of the final clinical resource is available in Additional file 3.

#### Resultant changes to the implementation intervention

Changes were made to the draft implementation intervention to address the identified barriers to using the clinical resource. Although not specifically tested in this study, the GP training session was modified to incorporate feedback from GPs and health consumers. Changes to GP training included: 1) emphasising the importance of individualising the resource to the patients; 2) emphasising that patients are likely to continue to refer to the resource after the consult; 3) providing suggested methods of using the resource in practice; 4) informing GPs of certain patient characteristics that may result in patients being more or less likely to use the resource; and 5) providing suggestions for storage of the resource in a conspicuous location to aid recall and use.

Consideration was given to whether an electronic version of the resource should be developed, but it was decided that it was not practical at this stage. Given consumers strongly favoured a hardcopy booklet it was decided to continue with the booklet version and test feasibility of use in clinical practice. While not addressed at this stage, cost of printing of the resource, and keeping the material updated also need to be considered, prior to broad implementation in clinical practice.

### Final implementation intervention to reduce GP referral for non-indicated imaging for LBP

The final implementation intervention, after development and modifications from preliminary testing, comprises of:

1. A developed clinical resource in the form of a LBP management and education booklet (PDF available in Additional file 3) designed to:
  Provide clinical decision support to the GPs
  Provide a reminder to GPs of appropriate clinical indicators for imaging of the low back
  Facilitate communication between GPs and patients to provide reassurance and explain why imaging isn’t required in their case
  Provide the GP with a useful clinical resource that they can give the patient to take home instead of a non-indicated imaging referral
  Provide the patient with a resource, individualised for them by the GP, to include information on: why the GP determined they didn’t need imaging, what management strategies they should undertake, and what to do if their LBP does not resolve
  Provide the patient with educational resources they can continue to refer to, and share with friends or family
  Be quick, easy, and appealing to read
2. GP training session with a trained facilitator (outline available in Additional file 4) designed to:
  Educate GPs on the appropriate use of imaging through discussion and the provision of published resources [4, 38]
  Explain why the clinical resource was developed and how it is intended to be used through discussion, provision of an information sheet (available in Additional file 5), and demonstration of how to use the clinical resource in clinical practice

## Discussion

This study used the Behaviour Change Wheel, informed by current evidence and stakeholder feedback, to develop an implementation intervention targeting both GP and patient behaviours concurrently, with the aim of reducing non-indicated imaging in patients with LBP. The resultant implementation intervention includes: 1) GP use of a developed clinical resource during a consult for LBP to facilitate patient management without referring for non-indicated imaging, and 2) a GP training session to provide GP education on appropriate indicators of imaging, and demonstrate the intended use of the clinical resource. Facilitators and barriers to the use of the resource in clinical practice were identified, and where possible, the implementation intervention was modified accordingly. This included alteration to wording within the resource identified as potentially confusing, and the delivery of additional information during GP training. GPs and health consumers thought the clinical resource would be beneficial to clinical practice. Health consumers reported that use of the resource was likely to make them more accepting of the GP decision not to image.

Systematic use of the Behaviour Change Wheel, with integration of the Theoretical Domains Framework, allowed for a structured approach to development of an implementation intervention informed by prior research. Previously, most interventions aiming to reduce imaging for LBP have attempted to improve GP knowledge of appropriate imaging referral, however, little evidence of change in imaging referral rates has been observed [16]. Using the Behaviour Change Wheel it was determined that both GP and patient related barriers need to be addressed to facilitate GP ability to manage the clinical consult without referring for non-indicated imaging, and decrease pressure from patients to refer for imaging. Use of the Behaviour Change Wheel led to the determination of key domains requiring behaviour change in both GPs and patients, including: Knowledge; Memory, attention, and decision process; and Environmental context and resources. Resultant mapping of behavioural change techniques led to the development of an evidence-informed and targeted implementation intervention. Further strengthening this study, preliminary testing was performed, with feedback from LBP experts, GPs and health consumers resulting in key changes to the final implementation intervention.

Limitations of this study include the inability to address all identified barriers to reducing imaging for LBP. Potential strategies to reduce barriers within the health care system, such as inadequate referral systems and pressure from external or third party payers (i.e. insurance payments) [46–51] did not meet the APEASE criteria as defined in the Behaviour Change Wheel process, as they would require government or systems level changes.

Not all identified barriers from the various stakeholders could be addressed due to a lack of practicability and acceptability. GPs reported that the ability to store and remember to use the clinical resource as a hardcopy booklet was a barrier to use, and an electronic printable version was suggested as a better option. However, the resource would not easily translate into a printable document, and would require removal of key components seen as integral to the intervention by both GPs and health consumers, such as the clinical decision support and the individualised management plan. Furthermore, health consumers reported that they would be much more likely to accept and use the resource as a hardcopy booklet compared to a printed handout, producing a discrepancy that could not be immediately resolved amongst the stakeholders. Feasibility testing with the resource as a hardcopy booklet is planned prior to future effectiveness testing, to assess whether GPs will use it as trained.

Printing costs and ongoing currency of the clinical resource were also raised as potential barriers to use. While not the focus of this study, consideration is needed about how the clinical resource will be maintained and distributed, and who will meet the associated ongoing costs when moving into future feasibility and effectiveness testing prior to large-scale implementation.

Finally, some health consumers reported that reading the clinical resource did not decrease their desire for imaging. In this study, to assess the appropriateness of the clinical resource content and its format, the health consumers were only provided with the clinical resource to read without any interaction with a GP. It is likely that the combination of GP explanation with reading the clinical resource will be more effective in educating patients than patients simply reading the resource alone. Some GPs also reported that they did not feel the clinical resource would be appropriate for all patients. The clinical resource has not been designed for use with all LBP patients. Some patients may require imaging to optimise management of their LBP, and some patients may respond well to GP advice and not require additional resources. Although the clinical resource may not be used with all LBP patients, using it with those patients who need more education or reassurance is likely to reduce rates of non-indicated imaging for LBP. Future feasibility and effectiveness testing will be used to assess how the implementation intervention is used in practice, and whether it is effective in reducing non-indicated imaging for LBP.

Two other studies have used behaviour change theory, incorporating the Theoretical Domains Framework, to develop an intervention to improve management of LBP [30, 52] with varied evidence of effectiveness [52, 53]. Both of these studies addressed overuse of imaging as one component of LBP management rather than as the primary focus. Similar barriers and facilitators to the current study were identified, however, patient related barriers were not specifically addressed and the focus of the interventions was on GP education. French et al. (2013) included a patient education handout within the intervention [30]. However, this was not an interactive, purpose-designed resource to aid GP ability to manage LBP without the use of non-indicated imaging, as in the current study.

## Conclusion

Behaviour change theory and preliminary testing were used to develop an implementation intervention to reduce non-indicated imaging for LBP in general medical practice. The implementation intervention includes: 1) GP use of a developed clinical resource during a consult for LBP to facilitate patient management without the use of non-indicated imaging, and 2) a GP training session to provide GP education on appropriate indicators of imaging, and demonstrate the intended use of the resource. Feasibility and pilot testing now needs to be conducted on the intervention prior to future effectiveness testing.

## Additional files

Additional file 1: Selection of appropriate intervention options. Mapping of the Com-B components and the Theoretical Domains Framework to intervention functions and policy categories that meet the APEASE criteria. (DOCX 15 kb)

Additional file 2: Barriers and facilitators to using the booklet. Barriers and facilitators to the use of the second iteration of the developed clinical resource in clinical practice. (DOCX 17 kb)

Additional file 3: Patient education booklet. PDF copy of the final clinical resource: LBP education and management booklet. (PDF 797 kb)

Additional file 4: Outline of GP training. Outline of GP training session. (DOCX 17 kb)

Additional file 5: GP information sheet. (PDF 260 kb)

## Abbreviations

APEASE: Affordability, practicability, effectiveness and cost-effectiveness, acceptability, side-effects and safety, equity
COM-B: Capability, opportunity, motivation, behaviour
GP: General medical practitioner
LBP: Low back pain
MUHREC: Macquarie University human research ethics committee

## References

[1] Hoy D, Brooks P, Blyth F, Buchbinder R (2010). The epidemiology of low back pain. Best Pract Res Clin Rheumatol.

[2] Hoy D, March L, Brooks P, Blyth F, Woolf A, Bain C, Williams G, Smith E, Vos T, Barendregt J (2014). The global burden of low back pain: estimates from the global burden of disease 2010 study. Ann Rheum Dis.

[3] Traeger A, Buchbinder R, Harris I, Maher C (2017). Diagnosis and management of low-back pain in primary care. CMAJ.

[4] Maher C, Underwood M, Buchbinder R (2017). Non-specific low back pain. Lancet.

[5] Chou R, Deyo RA, Jarvik JG (2012). Appropriate use of lumbar imaging for evaluation of low back pain. Radiol Clin N Am.

[6] Hong AS, Ross-Degnan D, Zhang F, Wharam JF (2017). Clinician-level predictors for ordering low-value imaging. JAMA Intern Med.

[7] Kost A, Genao I, Lee JW, Smith SR (2015). Clinical decisions made in primary care clinics before and after choosing wiselyTM. J Am Board Fam Med.

[8] Clinician lists. Recommendations for low back pain [http://www.choosingwisely.org/clinician-lists/#keyword=low_back_pain]. Accessed 12 Jan 2018.

[9] Darlow Ben, Forster Bruce B, O'Sullivan Kieran, O'Sullivan Peter (2016). It is time to stop causing harm with inappropriate imaging for low back pain. British Journal of Sports Medicine.

[10] Rao JK, Kroenke K, Mihaliak KA, Eckert GJ, Weinberger M, Rao JK, Kroenke K, Mihaliak KA, Eckert GJ, Weinberger M (2002). Can guidelines impact the ordering of magnetic resonance imaging studies by primary care providers for low back pain?. Am J Manag Care.

[11] Emery DJ, Shojania KG, Forster AJ, Mojaverian N, Feasby TE (2013). Overuse of magnetic resonance imaging. JAMA Intern Med.

[12] Muntion-Alfaro MT, Benitez-Camps M, Bordas-Julve JM, De Gispert-Uriach B, Zamora-Sanchez V, Galindo-Parres C (2006). Back pain: do we follow the recommendations of the guidelines?. [Spanish]. Aten Primaria.

[13] Gonzalez-Urzelai V, Lopez-de-Munain J (2003). Routine primary care management of acute low back pain: adherence to clinical guidelines. Eur Spine J.

[14] Kennedy SA, Fung W, Malik A, Farrokhyar F, Midia M (2014). Effect of governmental intervention on appropriateness of lumbar MRI referrals: a Canadian experience. J Am Coll Radiol.

[15] Slade SCP, Kent PP, Patel SDP, Bucknall TP, Buchbinder RP (2016). Barriers to primary care clinician adherence to clinical guidelines for the Management of low Back Pain: A Systematic Review and Meta-synthesis of Qualitative Studies. Clin J Pain.

[16] Jenkins HJ, Hancock MJ, French SD, Maher CG, Engel RM, Magnussen JS (2015). Effectiveness of interventions designed to reduce the use of imaging for low-back pain: a systematic review. Can Med Assoc J.

[17] Jenkins HJ, Hancock MJ, Maher CG, French SD, Magnussen JS (2016). Understanding patient beliefs regarding the use of imaging in the management of low back pain. Eur J Pain.

[18] Werner EL, Ihlebaek C, Skouen JS, Laerum E (2005). Beliefs about low back pain in the Norwegian general population: are they related to pain experiences and health professionals?. Spine.

[19] Hoffmann Tammy C (2013). Patients’ expectations of acute low back pain management: implications for evidence uptake. BMC Fam Pract.

[20] Wilson I, Dukes K, Greenfield S, Kaplan S, Hillman B (2001). Patients’ role in the use of radiology testing for common office practice complaints. Arch Intern Med.

[21] Espeland A, Baerheim A, Albrektsen G, Korsbrekke K, Larsen J (2001). Patients’ views on importance and usefulness of plain radiography for low Back pain. Spine.

[22] Buchbinder R (2001). Population based intervention to change back pain beliefs and disability: three part evaluation. BMJ Br Med J.

[23] Gross D, Russell A, Ferrari R, Battie M, Schopflocher D, Hu R, Waddell G, Buchbinder R (2010). Evaluation of a Canadian back pain mass media campaign. Spine.

[24] Werner EL, Ihlebaek C, Laerum E, Wormgoor M, Indahl A (2008). Low back pain media campaign: no effect on sickness behaviour. Patient Educ Couns.

[25] Waddell G (2007). Working backs Scotland: a public and professional health education campaign for back pain. Spine (Philadelphia 1976).

[26] Burton AK (1999). Information and advice to patients with back pain can have a positive effect - a randomized controlled trial of a novel educational booklet in primary care. Spine (Philadelphia 1976).

[27] George SZ, Teyhen DS, Wu SS, Wright AC, Dugan JL, Yang G, Robinson ME, Childs JD (2009). Psychosocial education improves low back pain beliefs: results from a cluster randomized clinical trial (NCT00373009) in a primary prevention setting. Eur Spine J.

[28] Michie S, Atkins L, West R (2014). The behaviour change wheel. A guide to designing interventions.

[29] Michie S, van Stralen MM, West R (2011). The behaviour change wheel: a new method for characterising and designing behaviour change interventions. Implement Sci.

[30] French SGS, O'Connor D, McKenzie J, Francis J, Michie S, Buchbinder R, Schattner P, Spike N, Grimshaw J (2012). Developing theory-informed behaviour change interventions to implement evidence into practice: a systematic approach using the theoretical domains framework. Implement Sci.

[31] Hodder RK, Wolfenden L, Kamper SJ, Lee H, Williams A, O'Brien KM, Williams CM (2016). Developing implementation science to improve the translation of research to address low back pain: a critical review. Best Pract Res Clin Rheumatol.

[32] Bartholomew LK, Parcel GS, Kok G (1998). Intervention mapping: a process for developing theory and evidence-based health education programs. Health Educ Behav.

[33] Craig P, Dieppe P, Macintyre S, Michie S, Nazareth I, Petticrew M (2008). Developing and evaluating complex interventions: the new Medical Research Council guidance. BMJ.

[34] Michie S, Johnston M, Francis J, Hardeman W, Eccles M (2008). From theory to intervention: mapping theoretically derived behavioural determinants to behaviour change techniques. Appl Psychol.

[35] Mesner SA, Foster NE, French SD (2016). Implementation interventions to improve the management of non-specific low back pain: a systematic review. BMC Musculoskelet Disord.

[36] Chou R, Qaseem A, Owens D, Shekelle P (2011). Diagnostic imaging for low back pain: advice for high-value health care from the American College of Physicians. Ann Intern Med.

[37] Chou R, Qaseem A, Snow V, Casey D, Cross TJ, Shekelle P, Owens DK (2007). Diagnosis and treatment of low back pain: a joint clinical practice guideline from the American college of physicians and the American pain society. Ann Intern Med.

[38] Qaseem A, Wilt TJ, McLean RM, Forciea MA (2017). Noninvasive treatments for acute, subacute, and chronic low Back pain: a clinical practice guideline from the American college of PhysiciansNoninvasive treatments for acute, subacute, and chronic low Back pain. Ann Intern Med.

[39] Maher CG, Williams C, Lin C, Latimer J (2011). Managing low back pain in primary care. Aust Prescr.

[40] Burton K, Klaber Moffett J, Main C, Roland M, Waddell G (2002). The Back book.

[41] Acute Low Back Pain [https://www.nhmrc.gov.au/guidelines-publications/cp94-cp95]. Accessed 3 Mar 2015.

[42] Advice for managing low back pain [http://www.sahealth.sa.gov.au/wps/wcm/connect/a61c510049e4d938b3aefb3a89b74631/ManagingLowBackPain-RAH-AlliedHealth-120123.pdf?MOD=AJPERES&CACHEID=ROOTWORKSPACE-a61c510049e4d938b3aefb3a89b74631-lmsDqWI]. Accessed 3 Mar 2015.

[43] Scans and low back pain [http://www.sahealth.sa.gov.au/wps/wcm/connect/1227450049e4e01cb4bffe3a89b74631/ScansAndLowBackPain-RAH-AlliedHealth-120123.pdf?MOD=AJPERES&CACHEID=ROOTWORKSPACE-1227450049e4e01cb4bffe3a89b74631-llRyqiC]. Accessed 3 Mar 2015.

[44] Hodges P, Nielsen A, French S (2015). Key messages for patients with low back pain: expert and consumer opinion. Physiotherapy.

[45] Verbeek J, Sengers M-J, Riemens L, Haafkens J (2004). Patient expectations of treatment for back pain: a systematic review of qualitative and quantitative studies. Spine.

[46] Espeland A, Baerheim A (2003). Factors affecting general practitioners’ decisions about plain radiography for back pain: implications for classification of guideline barriers–a qualitative study. BMC Health Serv Res.

[47] Fullen B, Doody C, Baxter GD, Daly L, Hurley D (2008). Chronic low back pain: non-clinical factors impacting on management by Irish doctors. Ir J Med Sci.

[48] Sears ED, Caverly TJ, Kullgren JT (2016). Clinicians’ perceptions of barriers to avoiding inappropriate imaging for low back pain— knowing is not enough. JAMA Intern Med.

[49] Schers H, Wensing M, Huijsmans Z, van Tulder M, Grol R (2001). Implementation barriers for general practice guidelines on low back pain: a qualitative study. Spine.

[50] Shye DFD, Romeo J, Eraker S (1998). Understanding physicians’ imaging test use in low back pain care: the role of focus groups. Int J Qual Health Care.

[51] Baker R, Lecouturier J, Bond S (2006). Explaining variation in GP referral rates for x-rays for back pain. Implement Sci.

[52] Lin IB, Coffin J, O’Sullivan PB (2016). Using theory to improve low back pain care in Australian aboriginal primary care: a mixed method single cohort pilot study. BMC Fam Pract.

[53] French S, McKenzie J, O'Connor D, Grimshaw J, Mortimer D, Francis J, Michie S, Spike N, Schattner P, Kent P (2013). Evaluation of a theory-informed implementation intervention for the Management of Acute low Back Pain in general medical practice: the IMPLEMENT cluster randomised trial. PLoS One.

[54] Dahan R, Borkan J, Brown JB, Reis S, Hermoni D, Harris S (2007). The challenge of using the low back pain guidelines: a qualitative research. J Eval Clin Pract.

[55] Slade SC, Kent P, Bucknall T, Molloy E, Patel S, Buchbinder R (2015). Barriers to primary care clinician adherence to clinical guidelines for the management of low back pain: protocol of a systematic review and meta-synthesis of qualitative studies. BMJ Open.

[56] Stafford VGS, Davidson I (2013). Why do patients with simple mechanical low back pain seek urgent care?. Physiotherapy.

