# Supplementary material for: Using behaviour change theory and preliminary testing to develop an implementation intervention to reduce imaging for low back pain
Source: BMC Health Serv Res. 2018 Sep 24;18:734. doi: 10.1186/s12913-018-3526-7 (PMC6154885; doi:10.1186/s12913-018-3526-7)
Supplement: Supplementary file 1 — Selection of appropriate intervention options. Mapping of the Com-B components and the Theoretical Domains Framework to intervention functions and policy categories that meet the APEASE criteria. (DOCX 15 kb) [file 12913_2018_3526_MOESM1_ESM.docx]

Additional file 1: Mapping of the Com-B components and the Theoretical Domains Framework to intervention functions and policy categories that meet the APEASE criteria

| **COM-B* component** | **Theoretical Domains Framework** | **Intervention function^ᵻ^ (meets APEASE^#^ criteria Y/N)** | **Policy Category^ⱡ^ (meets APEASE^#^ criteria Y/N)** |
| --- | --- | --- | --- |
| Physical capability | Physical skills | Training (Y) | Service provision (Y)  Guidelines (N)  Fiscal measures (N)  Regulation (N)  Legislation (N) |
| Psychological capability | Knowledge | Education (Y) | Communication/marketing (Y)  Service provision (Y)  Guidelines (N)  Regulation (N)  Legislation (N) |
| Psychological capability | Memory, attention, and decision process | Training (Y)  Environmental restructuring (Y)  Enablement (Y) | Service provision (Y)  Environmental/social planning (Y)  Guidelines (N)  Fiscal measures (N)  Regulation (N)  Legislation (N) |
| Physical opportunity | Environmental context and resources | Training (Y)  Environmental restructuring (Y)  Enablement (Y)  Restriction (N) | Service provision (Y)  Environmental/social planning (Y)  Guidelines (N)  Fiscal measures (N)  Regulation (N)  Legislation (N) |
| Social opportunity | Social influences | Environmental restructuring (Y)  Modelling (Y)  Enablement (Y)  Restriction (N) | Service provision (Y)  Environmental/social planning (Y)  Guidelines (N)  Fiscal measures (N)  Regulation (N)  Legislation (N) |
| Reflective motivation | Beliefs about capabilities | Education (Y)  Persuasion (Y)  Modelling (Y)  Enablement (Y) | Communications/marketing (Y)  Service provision (Y)  Environmental/social planning (Y)  Guidelines (N)  Fiscal measures (N)  Regulation (N)  Legislation (N) |
| Reflective motivation | Beliefs about consequences | Education (Y)  Persuasion (Y)  Modelling (Y) | Communication/marketing (Y)  Service provision (Y)  Guidelines (N)  Regulation (N)  Legislation (N) |
| Automatic motivation | Emotion | Persuasion (Y)  Modelling (Y)  Enablement (Y)  Incentivisation (N)  Coercion (N) | Communications/marketing (Y)  Service provision (Y)  Environmental/social planning (Y)  Guidelines (N)  Fiscal measures (N)  Regulation (N)  Legislation (N) |

Key:

*COM-B: Capability, Opportunity, Motivation, and Behaviour; as defined in the Behaviour Change Wheel

**^ᵻ^**Intervention function: means by which an intervention will change behaviour; as defined in the Behaviour Change Wheel

^#^APEASE: Affordability, Practicability, Effectiveness and cost-effectiveness, Acceptability, Side-effects and safety, Equity; as defined in the Behaviour Change Wheel

**^ⱡ^**Policy category: means by which an intervention will be delivered; as defined in the Behaviour Change Wheel

**Reasons APEASE criteria not met**

**Intervention functions:**

Restriction: Enforced restriction to GP ability to refer for non-indicated imaging is limited due to lack of: 1) Practicability (lack of suitable diagnostic criteria); 2) Acceptability (GP acceptance of limitation to referral rights); and 3) Safety (lack of suitable diagnostic criteria may miss cases requiring imaging)

Incentivisation: The use of incentives to limit GP referral for non-indicated imaging is limited due to lack of: 1) Affordability (ability of the research team to provide monetary incentives); 2) Practicability (ability of the research team to produce health-care or government level changes to provide incentives); and 3) Safety (incentives may lead to non-imaging when required)

Coercion: Creating an expectation of punishment for GP’s if they refer for non-indicated imaging is limited due to lack of: 1) Practicability (lack of suitable diagnostic criteria); 2) Acceptability (GP acceptance of this possibility); and 3) Safety (lack of suitably specific diagnostic criteria may lead to failure to image when required)

**Policy categories:**

Guidelines: The creation of guidelines is not required as guidelines currently exist, and new guidelines are not currently indicated. Distribution and education of current guidelines will be used within the developed implementation intervention.

Fiscal measures: Using the tax system to impact the financial cost is not indicated due to a lack of: 1) Practicability (ability of the research team to change fiscal measures); and 2) Acceptability (government, GPs and health consumers may not accept change).

Regulation: Rules or principles of practice behaviour are currently evident, however, not routinely adhered to. The aim of this research is to increase adherence to current regulation.

Legislation: Enforced restriction to GP ability to refer for non-indicated imaging through legislation is limited due to lack of: 1) Practicability (ability of the research team to change legislation); 2) Acceptability (GP acceptance of limitation to referral rights); and 3) Safety (enforcement of diagnostic criteria with limited specificity may miss cases requiring imaging)
