# Supplementary material for: Using behaviour change theory and preliminary testing to develop an implementation intervention to reduce imaging for low back pain
Source: BMC Health Serv Res. 2018 Sep 24;18:734. doi: 10.1186/s12913-018-3526-7 (PMC6154885; doi:10.1186/s12913-018-3526-7)
Supplement: Supplementary file 2 — Barriers and facilitators to using the booklet. Barriers and facilitators to the use of the second iteration of the developed clinical resource in clinical practice. (DOCX 17 kb) [file 12913_2018_3526_MOESM2_ESM.docx]

Additional file 2: Barriers and facilitators to the use of the second iteration of the developed clinical resource in clinical practice

| **Theme** | **General practitioner** | | **Laypeople (with a history of low back pain)** | |
| --- | --- | --- | --- | --- |
|  | **Barriers** | **Facilitators** | **Barriers** | **Facilitators** |
| **Format of the booklet** | **Hardcopy format of booklet - cost**  “if I had to pay for this booklet to have in my practice I’d probably have a few of them just to have in case or one for each doctor and then I’d just do a copy and printout like the one you sent me” (GP8)  **Hardcopy format of booklet – maintaining currency**  “it [hard copy] might go out of date in 10 years time” (GP4) | **Hardcopy format of booklet – easier to use**  “I think it’s probably easier to give out a hard copy [booklet] and we don’t have email at our surgery so I wouldn’t be emailing patients a link or anything” (GP1) | **Hardcopy format of booklet – unlikely to use further resources**  “I probably wouldn’t use them [links to low back pain sources] because I have to type something in. I’m more likely to google at that point” (MoP7) | **Hardcopy format of booklet – more useful**  “I think it works best as a booklet, if it’s one piece of paper are you going to be able to fit all this information in because this is 4 bits of paper and I think you need everything in there” (MoP4) |
|  |  |  |  |  |
|  | **Electronic format of booklet – email not useful**  “I wouldn’t [email a PDF], I know others would but I tend not to give my direct email address to a patient because then I get bombarded with ridiculous emails in the future” (GP10) | **Electronic format of booklet – easier to use A4 printout**  “If I could print it out yes [more likely to hand out A4 information sheet than booklet], so I’m always looking for good summaries that are nice and simple for patients” (GP10)  **Electronic format of booklet – email useful**  “yes that would be great too [online version, email to patient]” (GP3) | **Electronic format of booklet – unlikely to use A4 printout**  “I prefer this way. We get lots of those A4 print outs whenever we go to the doctor and I do review them and then I take away the pieces of information that I need, but there’s something about this and the management plan, like to know that you’ve got something to go through in the future” (MoP7)  **Electronic format of booklet – unlikely read email**  “Email form for me gets sorted and not necessarily revisited again and web format probably similar to email where it’s not really attended to on a secondary or third basis” (MoP6) | **Electronic format of booklet – likely to use**  “Yes [an email copy would be useful too]; I guess I prefer most things to be by email now certainly email would help for the additional resources” (MoP2) |
|  |  |  |  |  |
| **Usefulness of the booklet** | **Uncertainty of usefulness of booklet in all patients**  “You know so many people have a fixed idea about what they expect and walk in expecting things [from a GP appointment]; Other people would be more receptive [to receiving the booklet]” (GP4)  “It’s difficult to say how, you know people are very polite. You know you think they might but whether or not they’re going to read it I don’t know” (GP1) | **Belief in the usefulness of the booklet**  “if you actually give them a booklet that says that they don’t need it in the absence of the red flags then I think they’ll be more convinced” (GP9)  “there’s a need for this information to be made available to patients generally so I think they would receive it well” (GP6) | **Uncertainty of usefulness of booklet in all patients**  “maybe a bit one-sided, maybe there is, even though, people know there is times when you should get an xray” (MoP9)  “if I walked in there and I was in agony I’m not really sure if I want a booklet given to me and go home and read it” (MoP10) | **Belief in the usefulness of the booklet**  “the booklet would encourage me for one that I need to listen to the doctor at the end of the day and him telling me that well you don’t really need the xray mate or you do, and only that person can tell me that and if the booklet and the GP are both on the same page here of course I’d use the booklet” (MoP10)  “Yes [likely to read]; Because I have back pain; It’s very professional so I liked the look of, like when I read it I feel like I’m not getting, like, inaccurate information, as opposed to when you like doctor google something and get um conflicting information” (MoP7)  “I did like the links you had at the back page I think that was really helpful I had a look at some of them and thought that they were useful resources” (MoP2) |
|  |  |  |  |  |
|  | **Don’t need the booklet**  “I didn’t think that I’d probably use it in my practice, possibly because I don’t usually get a lot of I guess pressure to do imaging with back pain so it doesn’t normally come up” (GP5) | **Low back pain is a difficult area to manage, with a need for resources**  “yes [remember to use booklet] absolutely partly because you know low back pain freaks me out; if I had it to hand I can think thank goodness I’ve just got that book so I can just bring it out and we can just go through it” (GP4)  “there’s a need for this information to be made available to patients generally so I think they would receive it well” (GP5) |  |  |
|  |  |  |  |  |
|  |  | **Useful with patients needing further reassurance and explanations**  “I think it would be the ones who are more needing something else too – to tell them that they don’t need imaging, so on an as needed basis, not with everyone” (GP7) |  |  |
|  |  |  |  |  |
|  |  | **Useful as an ongoing reference or reminder**  “The main times that I want to work through a piece of paper with someone is if I want to refresh my memory. So for example the red flags type of thing so if I want to fall back onto a check list because I want to reassure myself that I’m not missing anything.” (GP10) |  |  |
|  |  |  |  |  |
|  |  | **Useful to meet patient expectations**  “Yes I’d like to fill in these things as I think the patients feel it’s sort of individualised for them and it gives you a focus for discussing what treatments they can do themselves” (GP4)  “yeah I think that patients who leave without a piece of paper in their hand are usually less satisfied than those who leave with a piece of paper in their hand” (GP10) |  |  |
|  |  |  |  |  |
| **Use of the booklet in clinical practice** | **Too time consuming to use**  “there might be a bit more time expended during a consultation going through it in that way rather than kind of just going with the flow of the consultation” (GP2) | **Aids time-efficient delivery of information**  “[using in a time efficient manner] I think it’s actually good to have something like this because whatever I say there or even if I forget to say they will still have this thing to look into” (GP8) |  | **Time-efficient to read**  “the booklet’s not a big booklet so it’s not like you have to sit there and you need an hour to read it it’s only a good little 10 to 15 minute read” |
|  | **Unlikely to individualise the booklet**  “probably not filling it out but I’d probably go through it with them but I probably wouldn’t – depends, depends on how much time I have” (GP3) | **Would individualise the booklet**  “I’d probably use it [the booklet] to write things in or tick in, but probably not every patient” (GP3) |  | **Individualised advice**  “it [individualised options] just gives you that sort of idea that it’s more, well it’s personal to you rather than being just some information that the GPs dolling out” (MoP6)  “I don’t know, it’s definitely that thing about what they can write in it and then handing you something and taking you through it, it makes it more tangible and more tactile” (MoP4) |
|  |  |  |  |  |
|  | **Forgetting to use the booklet during a consult**  “I think the main thing [disadvantage] was the amount of resources in terms of maybe it would just get lost amongst all the other pamphlets and booklets that we do have” (GP7) | **Booklets available and accessible**  “I would [remember to use booklet] provided that I’ve got enough copies and it’s in an accessible area. If I had to run out to a different room to get the booklet and I’m in a rush I might not use it, so if it’s right in front of me on my desk then yes I would.” (GP9)  “I have a special box where I keep these kind of brochures and various things and I tend to pull them out. I also keep another one where there are folders where I keep print outs of these patient education material that I collected” (GP8)  **Electronic version**  “Yes I think on the computer its easier [to remember to use]” (GP3) |  |  |
|  |  |  |  |  |
|  |  | **Use the booklet to explain diagnosis and management**  “I’d just listen to their history and then do my examination so I knew what we’re dealing with and have a better idea and then I’d bring it [the booklet] out as I’m explaining what we’re going to do next” (GP4) |  | **Booklet more useful if reinforced by the GP**  “I think the booklet would help, but I think you actually need someone to sort of reinforce that information” (MoP1) |
|  |  |  |  |  |
|  |  | **Give the booklet to the patient to read only**  “I might just give it [the booklet] to them completely to read” (GP6) |  | **Happy to read the booklet at home**  “I’d be happy to take it home and read it but I suppose there would be some people who would like to have it explained to them but yeah no it’s all pretty easy for me to understand so I’d be happy to take it home and read it” (MoP8)  “I would think so yes [be likely to read it] it’s a short enough leaflet that you can read it very quickly without getting confused by excessive facts or being too long” (MoP3) |
|  |  |  |  | **Use booklet as an ongoing reference**  “having it written down about what you should do in the future so you don’t have to remember all the doctor said, that’s really good as I always forget everything” (MoP4)  “I think it might be something I personally will probably look at next time my back pain comes back and before I go to the doctor” (MoP5) |
