# Supplementary material for: Using behaviour change theory and preliminary testing to develop an implementation intervention to reduce imaging for low back pain
Source: BMC Health Serv Res. 2018 Sep 24;18:734. doi: 10.1186/s12913-018-3526-7 (PMC6154885; doi:10.1186/s12913-018-3526-7)
Supplement: Supplementary file 5 — GP information sheet. (PDF 260 kb) [file 12913_2018_3526_MOESM5_ESM.pdf]

DEPARTMENT OF HEALTH PROFESSIONS

Faculty of Medicine and Health Sciences

GP information sheet

UNDERSTANDING MY LOW BACK PAIN AND WHETHER I NEED IMAGING BOOKLET

The booklet 'Understanding my Low Back Pain and whether I need imaging' has been designed as a patient education resource for GPs to deliver during a standard clinical consult.

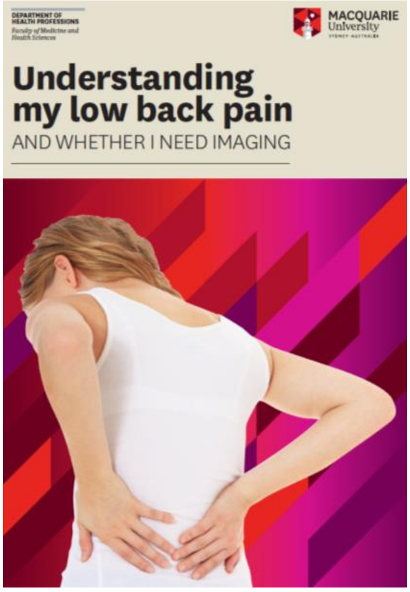

Who should the booklet be given to?

The booklet has been designed for patients with:

- Either a new episode or a recurrence (including a flare up of mild ongoing pain) of acute low back pain of less than 6 weeks duration
- No signs or symptoms of serious causes of low back pain (eg. fracture, infection, tumour or inflammatory arthritis) and no indications for low back surgery
- No current indications for imaging or further investigations

What are the aims of the booklet?

The aims of the booklet are to:

- Assist GPs to reassure patients that they are receiving the best management for low back pain without the use of imaging referrals
- Facilitate all elements of clinical consultation for low back pain including: screening, delivery of key messages and personalised management advice
- Provide the patient with further information to read from a list of trusted resources

How has the booklet been designed to be used?

Although this booklet could be used in many ways, it has been designed to be integrated into a standard consult:

- The patient is diagnosed with simple low back pain based on their GPs assessment
- The patient is shown the diagnosis algorithm on the first page. The GP checks the boxes and uses this to reassure the patient that they have simple low back pain and to explain imaging is not needed
- The GP can use the information in the booklet to help emphasise key messages about low back pain for each individual patient. The patient can read the rest of the booklet at home to increase time efficiency.
- The 'What is my low back pain management plan?' and 'What should I do next' sections are filled out as appropriate for the patient
- The patient is made aware of the extra resources available online before they are given the booklet
- The patient is able to use the booklet as an ongoing resource, for current and future episodes of low back pain

---

FIND OUT MORE
Hazel Jenkins
Macquarie University NSW 2109 Australia
T: +61 (2) 9850 9383
hazel.jenkins@mq.edu.au

CRICOS Provider 00002J
